# Randomized controlled trail of work-map: telehealth metacognitive intervention for work performance enhancement of adults with attention-deficit/hyperactivity disorder

**DOI:** 10.1192/j.eurpsy.2024.1142

**Published:** 2024-08-27

**Authors:** N. Grinblat, S. Rosenblum

**Affiliations:** Department of Occupational Therapy, Faculty of Social Welfare & Health Sciences, University of Haifa, Haifa, Israel

## Abstract

**Introduction:**

The literature has emphasized the importance of implementing evidence-based occupational therapy teleinterventions to enhance work participation in adults with attention-deficit/hyperactivity disorder (ADHD).

**Objectives:**

This study aimed to evaluate the efficacy of an innovative metacognitive self-tailored teleintervention for adults with ADHD performance at work enhancement (Work-MAP). The outcome measures were efficacy of and satisfaction with the performance of self-selected work goals (Canadian Occupational Performance Measure), executive functions (Behavior Rating Inventory of Executive Function-Adult), and quality of life (Adult ADHD Quality of Life Questionnaire).

**Methods:**

In this randomized controlled trial, participants were 46 adults with ADHD. Group A (*n* = 31) received the synchronous, hybrid-telehealth intervention in 11 weekly 1-hour individual sessions, while Group B (*n* = 15) completed the same intervention after a waiting phase.

**Results:**

Following the intervention, participants demonstrated and maintained significant improvements in all outcome measures (strong-to-moderate significant effects) to the 3-month follow-up.

**Conclusions:**

Work-MAP seems to be effective intervention for enhancing work participation (i.e., performance at work), executive functions, and quality of life of adults with ADHD. Future studies with larger samples and additional objective measures are needed to further validate these findings.

**Disclosure of Interest:**

None Declared

